# High-Aperture-Ratio Dual-View Integral Imaging Display

**DOI:** 10.3390/mi13122213

**Published:** 2022-12-14

**Authors:** Bai-Chuan Zhao, Fan Yang, Fei Wu

**Affiliations:** 1 School of Information Engineering, Chengdu Aeronautic Polytechnic, Chengdu 610218, China; 2 Chengdu Institute of Computer Application, Chinese Academy of Sciences, Chengdu 610041, China; 3 School of Electronic Engineering, Chengdu Technological University, Chengdu 610073, China

**Keywords:** high-aperture ratio, dual-view, integral imaging

## Abstract

Low aperture ratio is a problem in the conventional dual-view integral imaging (DVII) display using a point light source array. A high-aperture-ratio DVII display using a gradient width point light source array is reported in this work. The elemental Images 1 and 2, which are alternatively aligned on a liquid crystal panel, are illuminated by the light rays emitted from an assigned point light source. The optical path is optimized by optimizing the widths of the point light sources. The aperture ratio of the proposed DVII display was demonstrated as 1.88 times the conventional DVII display. Experiments showed that the vertical viewing range is related to the vertical width of the first row point light source, whereas the aperture ratio is related to the vertical widths of all point light sources. By optimizing the widths of the point light sources, the aperture ratio is enhanced without loss of viewing range.

## 1. Introduction

A dual-view display provides two two-dimensional (2D) images to multiple observers in different directions. In an automobile, the dual-view display provides Captain America for an adult passenger and Peppa Pig for a child passenger. Parallax barriers and lenticular lenses were used to split two 2D images in early dual-view display [1]. Patterned electrodes were fabricated to replace the parallax barriers and the lenticular lenses [2]. However, the dual-view displays mentioned above only provide 2D images.

Integral imaging display, which allows the observers to view true three-dimensional (3D) images, is considered a significant display [3,4,5,6,7,8,9,10,11]. The integral imaging display includes a pickup process and a display process. Two identical microlens arrays are used in the pickup and the display processes. However, the directions of the pickup and display processes are inverse. According to the reversibility of the optical path, the depth of the reconstructed images is reversed when compared to the original objects. Therefore, many pseudoscopic-to-orthoscopic (PO) conversion methods have been reported. In the early days, a two-step pickup method was proposed to realize PO conversion [12]. The second pickup process induced degradation of the 3D images. A gradient-index microlens array was adopted to replace the second pickup process [13]. The gradient-index microlens array was too expensive to be widely used. A practical PO conversion method was proposed [14]. The elemental images obtained in the pickup process are rotated by 180 degrees. The orthoscopic 3D scenes can be reconstructed by the rotated elemental images. Other methods have been studied to enlarge the applicable field by generating real and virtual 3D reconstructions [15,16,17]. A smart PO conversion approach virtually captures a reconstructed 3D image by using a virtual pinhole array [18]. However, these methods need extra computational load and cost time. The elemental images, the computational load, and the time have increased. To date, many methods have been provided to realize the dual-view integral imaging (DVII) display that provides two 3D images to multiple observers simultaneously [19,20,21,22]. There are two sets of elemental images in the DVII display. The computational load and time of the PO conversion in the DVII display are twice those in the II display. Therefore, the pseudoscopic problem hinders the application of the DVII display. Recently, a pyramid pinhole array was used to generate a point light source array without crosstalk [23]. The pseudoscopic problem was also resolved by reversing the direction of display process. Therefore, the point light source array was introduced in the DVII display [24]. In the DVII display based on the point light source array, there are display and non-display zones in the reconstructed 3D image. Therefore, the aperture ratio is used to evaluate the ratio of the display zone. When the aperture ratio is larger, the display effect is better. Increasing the width of the point light source can increase the aperture ratio while decreasing the viewing range. Therefore, the aperture ratio is limited in the DVII display based on the point light source array. Thus, we proposed a High-aperture ratio DVII display.

## 2. Structure and Method

**Figure 1:**
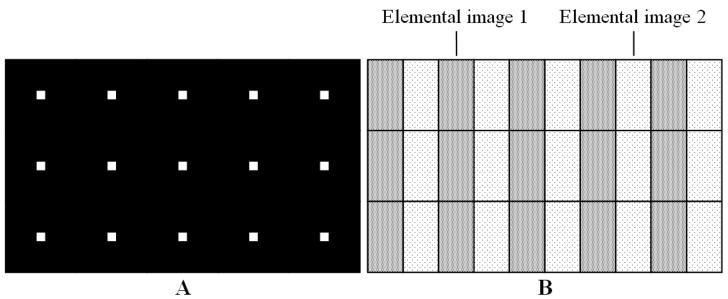
Structures of the conventional point light source array and the elemental image array.

The structures of the conventional point light source array (A) and the elemental image array (B) are shown in Figure 1. In the conventional point light source array, the horizontal and vertical widths are identical. Since the elemental Images 1 and 2 are alternatively displayed in the horizontal direction, the light rays through the elemental Images 1 and 2 are modulated into the left and right directions [24].

**Figure 2:**
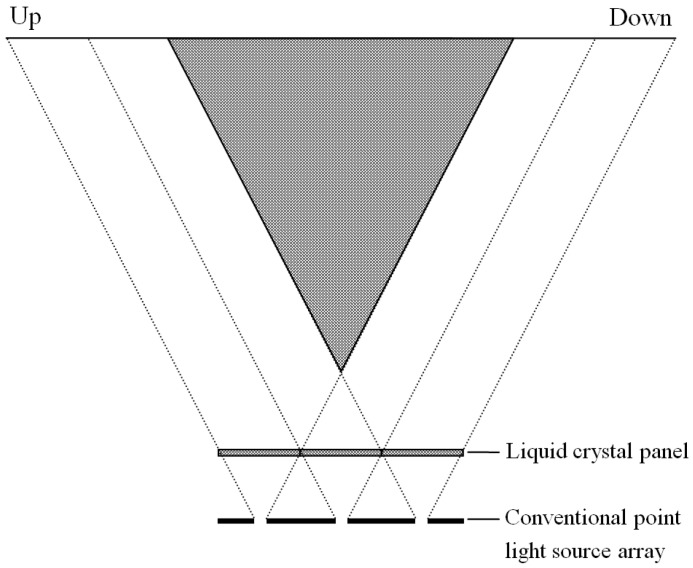
Principle of the conventional DVII display in vertical direction.

Because of the crosstalk of the adjacent point light sources, the light rays emitted from the left and right marginals of each point light source travel through the left and right marginals of the assigned elemental image, as shown in Figure 2 [24]. Since the light rays through the elemental images are parallel, the vertical viewing zones reconstructed by the elemental images partially overlap. The width of the vertical viewing zone is the public zone of all reconstructed vertical viewing zones. Therefore, the vertical viewing range of the conventional DVII display based on the point light source array is only decided by light rays emitted from the upmost and downmost point light sources. The other point light sources can be optimized to enhance the aperture ratio.

**Figure 3:**
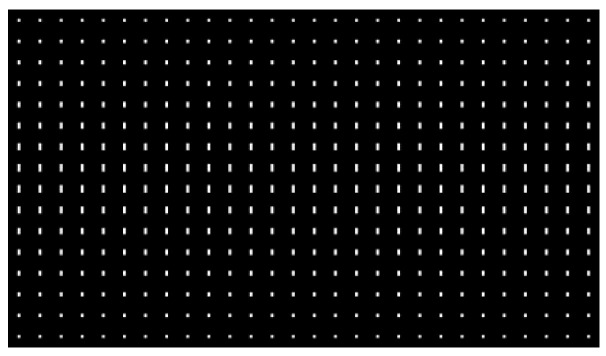
Structure of the gradient-width point light source array.

A gradient-width point light source array is proposed, as shown in Figure 3. In the gradient-width point light source array, the horizontal widths are identical, whereas the vertical widths are enlarged from both sides to the middle, as shown in Figure 3. 

**Figure 4:**
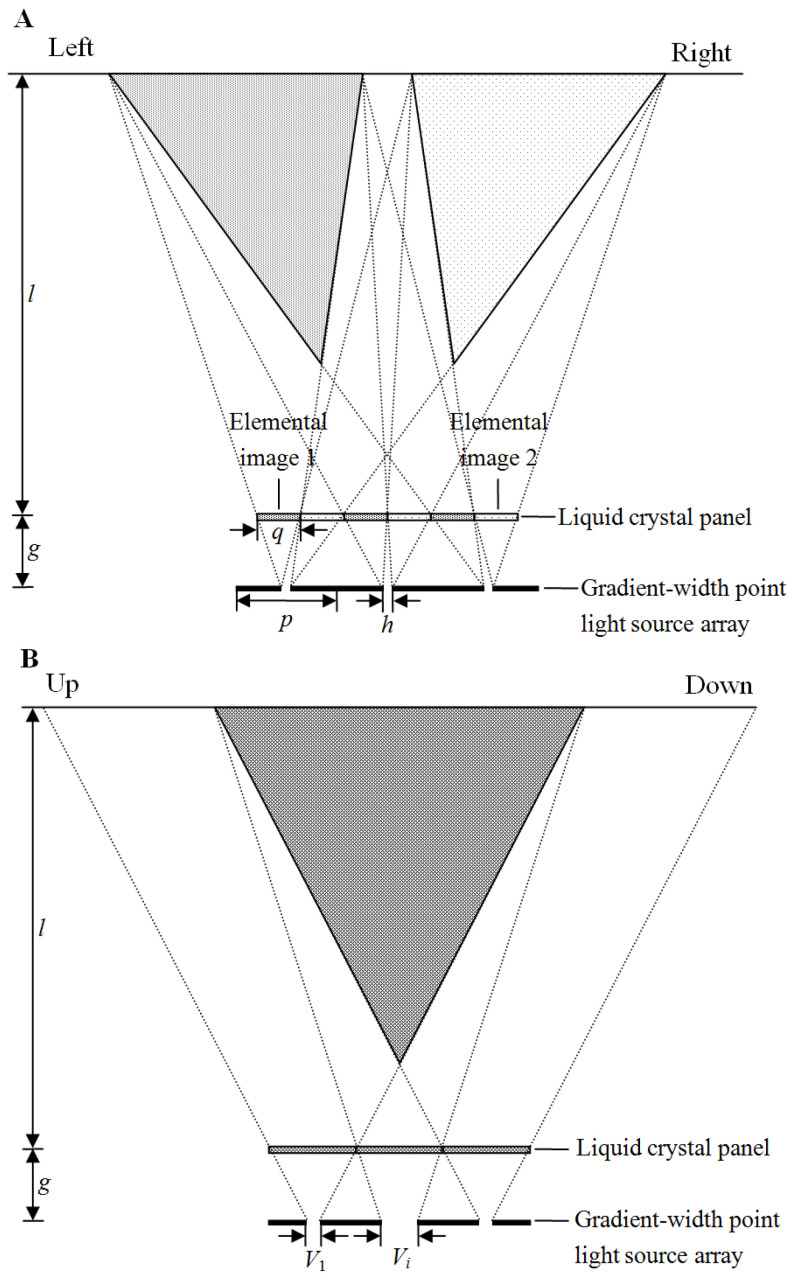
(**A**) Structure and light path in a horizontal direction. (**B**) Structure and light path in a vertical direction.

Figure 4 shows the structure of the proposed DVII display. It consists of the gradient-width point light source array and the liquid crystal panel. The elemental Images 1 and 2 on the liquid crystal panel are alternatively displayed in a horizontal direction. The horizontal pitch of elemental Image 1 is equal to that of elemental Image 2. The horizontal pitch of the point light source *p* is shown as

(1) $$p=\frac{2\left(l+g\right)q}{l},$$

where *q* means the horizontal pitch of the elemental image, *g* denotes the gap between the point light source and the elemental image, and *l* denotes the optimal viewing distance.

The light rays emitted from the point light source illuminate the assigned elemental Images 1 and 2. Similar to the conventional DVII display, the light rays emitted from the left and right marginals of each point light source pass through the left and right marginals of the assigned elemental image. Since the horizontal pitch of the point light source is optimized, the light rays through the elemental images are not parallel in the horizontal direction. At the optimal viewing distance, the light rays through elemental Images 1 coincide in the left direction, whereas the light rays through elemental Images 2 coincide in the right direction. The horizontal width of the left viewing zone is equal to the horizontal width of the reconstructed zone by each elemental Image 1, while the horizontal width of the right viewing zone is equal to the horizontal width of the reconstructed zone by each elemental Image 2. The horizontal viewing ranges *θ_L_* and *θ_R_* are shown as

(2) $$\theta_{L}\in \left\{-\arctan\left(\frac{2ql+2qg-hl}{2gl}\right),-\arctan\left(\frac{h}{2g}\right)\right\},$$

(3) $$\theta_{R}\in \left\{\arctan\left(\frac{h}{2g}\right),\arctan\left(\frac{2ql+2qg-hl}{2gl}\right)\right\},$$

where *h* denotes the horizontal width of the point light source, the negative sign indicates the left direction, and the positive sign indicates the right direction.

The vertical pitches of the point light source are equal to those of the elemental image. The vertical pitch of the elemental image is twice as much as the horizontal one. The light rays emitted from the up and down marginals of each point light source are through the up and down marginals of the assigned elemental image. Since the vertical widths of the point light sources are increased from both sides to the middle, the vertical widths of the reconstructed viewing zones are decreased from both sides to the middle. Similar to the conventional DVII display, the vertical viewing range of the proposed DVII display is also decided by light rays emitted from the upmost and downmost point light sources. In other words, although the point light sources are optimized, the crosstalk of the proposed DVII display is not enlarged, and the vertical viewing ranges of two 3D scenes are both identical to the conventional DVII display.

The light rays through the bottom of the elemental images on the upper half of the liquid crystal panel coincide, whereas the light rays through the top of the elemental images on the down half of the liquid crystal panel coincide. The vertical width of the *i*th row point light source *Vi* is deduced as

(4) $$\left\{ \begin{array}{l} V_{i}=V_{1}+\frac{4gq}{l+g}(i-1)\;\;\;\;\;\;1\leq i\leq \frac{n}{2},\\ V_{i}=V_{1}+\frac{4gq}{l+g}(n-i)\;\;\;\;\;\;\frac{n}{2}<i\leq n \end{array}\right.$$

where *V*_1_ denotes the vertical width of the first row point light source, and *n* denotes the number of point light sources in the vertical direction.

The vertical viewing ranges of two 3D scenes are identical. The vertical viewing range *θ_V_* is calculated as

(5) $$\theta_{V}\in \left\{-\arctan\left[\frac{\left(2q-V_{1}\right)l-2\left(n-2\right)qg}{2gl}\right],\arctan\left[\frac{\left(2q-V_{1}\right)l-2\left(n-2\right)qg}{2gl}\right]\right\},$$

where the negative sign means the up direction, and the positive sign means the down direction. The aperture ratio of the proposed DVII display *A* is shown as

(6) $$A=\sum_{i=1}^{n}\frac{hV_{i}}{4nq^{2}}$$

**Figure 5:**
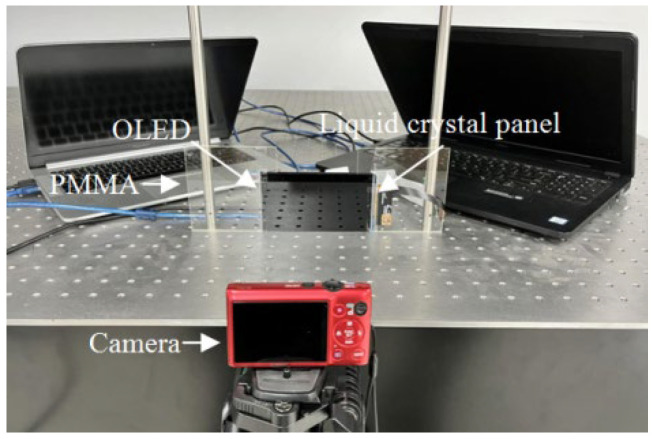
Experimental system of the proposed DVII display.

An experimental system was constructed to demonstrate the proposed structure, as shown in Figure 5. An OLED was introduced to display the gradient-width point light source array.

The resolution of the 5.5-inch OLED, whose model number is JYH055FR029, was 1920 × 1080. The resolution of the 5.5-inch liquid crystal panel, whose model number is FRD550L3901, was also 1920 × 1080. The sizes of the pixels on the OLED and the liquid crystal panel were both 0.063 mm. The luminance of the OLED was 700 cd/m^2^. The parameters of the experimental system are shown in Table 1.

**Figure 6:**
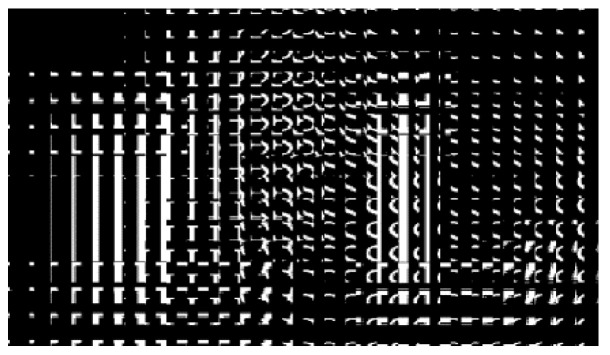
Elemental image array.

Letters “F” and “L” were captured in the left-view field, while letters “L” and “S” were captured in the right-view field using 3Ds Max software. The elemental Images 1 and 2 were combined by using MATLAB software. The elemental image array is shown in Figure 6. Each elemental image has 30 × 60 pixels.

## 3. Results

**Figure 7:**
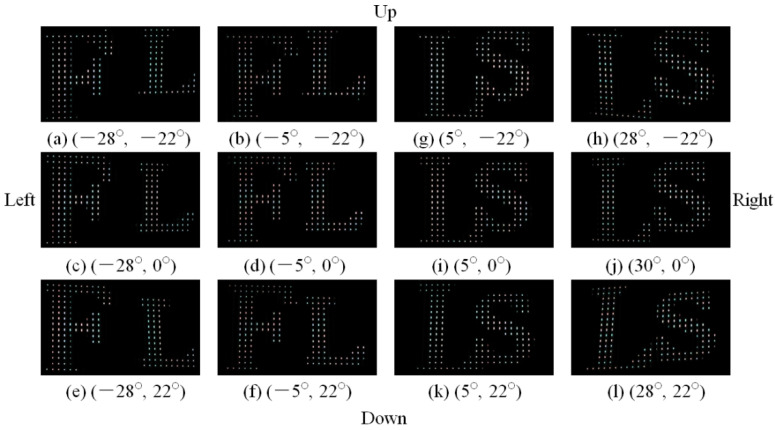
3D images presented by the proposed DVII display.

The 3D Scene 1 is provided into the left direction, as shown in Figure 7a–f. Letters “F” and “L” are seen from −28° to −5° in the horizontal direction. Letters “F” and “L” are captured from −22° to 22° in the vertical direction. The 3D Scene 2 is presented into the right direction, as shown in Figure 7g–l. Letters “L” and “S” are viewed from 5° to 28° in the horizontal direction. Letters “L” and “S” are exhibited from −22° to 22° in vertical direction. The relative gaps of letters “F” and “L” are changed with different angles. In other words, the reconstructed images obtained from different angles have relative displacement, indicating that the horizontal and vertical parallaxes are evident. The aperture ratio of the proposed DVII display was calculated as 3.3%.

**Figure 8:**
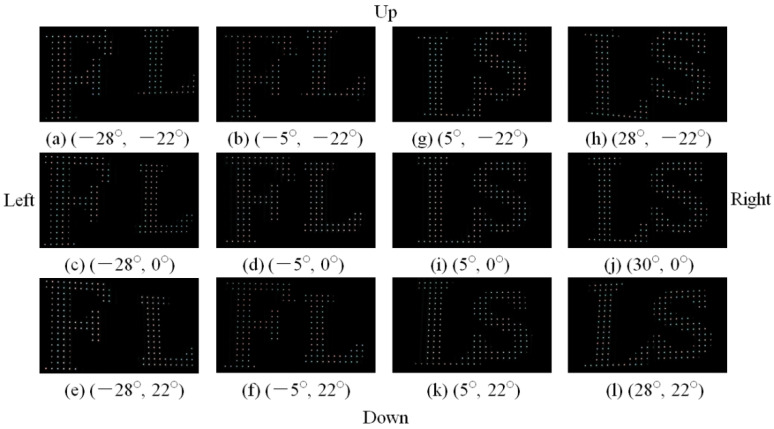
3D images presented by the conventional DVII display.

In comparison, the 3D Scenes 1 and 2 reconstructed in the conventional DVII display are shown in Figure 8. The width of the point light source was 0.252 mm. Letters “F” and “L” are seen from −28° to −5° in the horizontal direction, as shown in Figure 8a–f. Letters “F” and “L” are captured from −22° to 22° in the vertical direction. The 3D Scene 2 is presented into the right direction, as shown in Figure 8g–l. Letters “L” and “S” are viewed from 5° to 28° in the horizontal direction. The relative gaps of letters “L” and “S” are changed with different angles. In other words, the reconstructed images obtained from different angles have relative displacement, indicating that the horizontal and vertical parallaxes are also evident. Letters “L” and “S” are exhibited from −22° to 22° in vertical direction. The aperture ratio of the conventional DVII display was calculated as 1.7%.

## 4. Discussion

Compared with the conventional point light source array, the vertical widths in the gradient-width point light source array were optimized. The vertical viewing range was related to the vertical width of the first row point light source. Therefore, the aperture ratio was enhanced by increasing the vertical widths of other point light sources. The experimental results proved that the aperture ratio of the proposed DVII display was significantly higher than that of the conventional one, and the viewing ranges of the proposed DVII display were equal to those of the conventional one.

## Figures and Tables

**Table 1:** Parameters of the experimental system.

Parameters	Values
Horizontal pitch of the point light source	3.843 mm
Horizontal pitch of the elemental image	1.89 mm
Horizontal width of the point light source	0.5 mm
Vertical width of the first row point light source	0.5 mm
Gap between the OLED and the liquid crystal panel	3 mm
Optimal viewing distance	180 mm
Number of the point light sources in horizontal direction	28
Number of the point light sources in vertical direction	16

## Data Availability

Not applicable.
